# Indigenous Yeast Interactions in Dual-Starter Fermentations May Improve the Varietal Expression of Moschofilero Wine

**DOI:** 10.3389/fmicb.2019.01712

**Published:** 2019-07-26

**Authors:** Aspasia Nisiotou, Athanasios Mallouchos, Chrysoula Tassou, Georgios Banilas

**Affiliations:** ^1^Institute of Technology of Agricultural Products, Hellenic Agricultural Organization “Demeter”, Lykovryssi, Greece; ^2^Laboratory of Food Chemistry and Analysis, Department of Food Science and Human Nutrition, Agricultural University of Athens, Athens, Greece; ^3^Department of Wine, Vine and Beverage Sciences, University of West Attica, Athens, Greece

**Keywords:** non-*Saccharomyces*, wine fermentation, yeast starter cultures, *Hanseniaspora uvarum*, *Lachancea thermotolerans*, wine chemical profile, volatiles

## Abstract

Multi-starter wine fermentations employing non-*Saccharomyces* (NS) yeasts are becoming an emerging trend in winemaking. It is therefore important to determine the impacts of different NS strains in the wine phenotype and in particular the aroma outputs in different inoculation schemes and fermentation conditions. Here, two native NS yeasts, *Lachancea thermotolerans* LtMM7 and *Hanseniaspora uvarum* HuMM19, were assessed for their ability to improve the quality of Moschofilero, a Greek aromatic white wine. The NS strains were initially examined in laboratory scale fermentations in mixed inoculations with ScMM23, a native *Saccharomyces cerevisiae* strain. LtMM7 was selected to be further evaluated in pilot scale fermentations. Five different inoculation schemes were considered: single inoculation of ScMM23 (IS), simultaneous inoculation of ScMM23 with HuMM19 (SMH) or LtMM7 (SML), and sequential inoculation of HuMM19 (SQH) or LtMM7 (SQL) followed by ScMM23. At laboratory scale fermentations, the chemical profiles were largely affected by both the NS species and the inoculation scheme applied. The sequential inoculation using HuMM19 produced the most divergent wine phenotype. However, HuMM19 caused significant increases in acetic acid and ethyl acetate levels that impeded its use in pilot scale trials. LtMM7 significantly affected the chemical profiles of wines produced at the winery, especially in the sequential inoculation scheme. Importantly, LtMM7 significantly increased the levels of acetate esters or ethyl esters, depending on the inoculation method applied. In particular, acetate esters like isobutyl acetate, hexyl acetate, and 2-phenylethyl acetate, which all impart fruity or floral aromas, were significantly increased in SQL. On the other hand, higher levels of total ethyl esters were associated with SML. The most striking differences were observed in the levels of fruit-impair esters like ethyl decanoate, 3-methylbutyl octanoate, and isoamyl hexanoate. This is the first study to report a significant increase in the ethyl ester fraction by *L. thermotolerans*. Interestingly, *L. thermotolerans* in SQL also increased the concentrations of damascenone and geraniol, the major teprenic compound of Moschofilero, which are associated with several typical floral and fruity aromas of the variety. Present results show that *L. thermotolerans* may enhance the varietal character and increase the chemical complexity of Moschofilero wines.

## Introduction

During winemaking, fermentation of sugars is principally conducted by *Saccharomyces cerevisia*e, the major wine yeast. Although overwhelmed by non-*Saccharomyces* (NS) yeast populations in fresh must, *S. cerevisiae* quickly antagonizes other species and finishes the alcoholic fermentation. Its high efficiency to convert grape sugars to ethanol and the ability to withstand the harsh conditions of fermentation has established its hegemonic role in industrial winemaking as starter yeast culture (Albergaria and Arneborg, 2016). Typically, a selected *S. cerevisiae* strain is inoculated at high concentration immediately after grape berry crushing. However, by these means, the indigenous yeast microbiota is suppressed and the sensory profile of the wine is largely shaped by the inoculated strain. While the dominance of the starter culture ensures fermentation stability and reproducibility, it deprives wines of the organoleptic complexity or distinctiveness that a spontaneous fermentation may offer (Rainieri and Pretorius, 2000; Mannazzu et al., 2002). Therefore, there is a steadily increasing interest for the exploitation of NS yeasts, the so-called wild yeast flora, in winemaking.

Several yeast species are naturally found on grape skins and may develop during spontaneous alcoholic fermentation, particularly at the early stage of the course. Most of them belong to the genera *Hanseniaspora*, *Candida*, *Lachancea*, *Metschnikowia*, *Pichia*, *Torulaspora*, and *Zygosaccharomyces* (Nisiotou et al., 2007; Fleet, 2008). Although NS yeasts initiate the fermentation, most of them are not detectable at the end of the course, either because they are ethanol intolerant or incapable to withstand the microbial antagonism. However, their role is crucial for the production of important metabolites that shape the flavor and contribute to the style of wines (Jolly et al., 2014). It has been well established that diverse yeast species or strains may confer different characteristics to wines (Petruzzi et al., 2017; Sgouros et al., 2018). NS yeasts associated with the vineyard-ecosystem are characterized by high biodiversity that can be influenced by several factors, such as the grape cultivar, the sanitary status of grape berries, the viticultural practices, and environmental factors (Nisiotou and Nychas, 2007; Bokulich et al., 2014; Banilas et al., 2016; Drumonde-Neves et al., 2016; Guillamón and Barrio, 2017). Recent studies show that distant viticultural regions maintain different microbial communities (Gayevskiy and Goddard, 2012; Bokulich et al., 2014). Most importantly, such genetic differences may coincide well with phenotypic discrepancies among yeast populations that consequently generate discrete phenotypes and regional signatures in wines (Knight et al., 2015; Banilas et al., 2016; Nisiotou et al., 2018). Thus, the use of yeasts derived from a vineyard-ecosystem may enhance the typicity and genuineness of the respective local wines, bringing the vineyard microbiome to the fore as a new scale of the *terroir* concept, often referred to as microbial *terroir*.

During the last decade, there has been an increasing interest from the wine industry in the exploitation and utilization of NS wine yeasts. Accumulating data show that different NS species/strains may have interesting enological phenotypes for the formulation of new starter cultures (Roudil et al., 2019). Some examples include the production of mannoproteins, the lowering of volatile acidity or ethanol content, the production of various aroma compounds and color stabilization. *L. thermotolerans* appears as a promising candidate species for use as starter culture, due to several positive physiological characteristics that enhance the flavor and improve the overall quality of wine, such as the production of glycerol and 2-phenylethanol (Gobbi et al., 2013; Benito et al., 2016; Roudil et al., 2019). It has been also shown that certain strains can increase wine total acidity through the production of lactic acid while reducing the volatile acidity (Kapsopoulou et al., 2007; Banilas et al., 2016). Other positive enological attributes refer to the relatively high alcoholic fermentation ability and the low production of acetic acid by this species (Hranilovic et al., 2018). However, studies on the flavor profile of wines produced by mixed fermentations of *L. thermotolerans* and *S. cerevisiae* are limited (Balikci et al., 2016). *Hanseniaspora uvarum* (anamorph *Kloeckera apiculata*), another wild wine yeast that is typically encountered at the early stages of fermentation, has received much less attention as candidate NS-starter in winemaking. Actually, it has long been considered undesirable in winemaking, due to the high production of ethyl acetate that diminishes wine quality (du Toit and Pretorius, 2000; Ciani et al., 2006). However, recent studies have led to the reassessment of *H. uvarum* as conditionally beneficial for winemaking purposes (Albertin et al., 2016). It is probably the most abundant NS yeast in fresh must, along with *M. pulcherrima* (Fleet, 2003; Nisiotou and Nychas, 2007), and may largely affect wine character and typicity. Factually, it has been recently shown that selected *H. uvarum* strains can improve the organoleptic quality of wine aroma (Tristezza et al., 2016). When co-inoculated with S. cerevisiae produces chemically distinct wines compared to pure S. cerevisiae inocula, with increased flavor diversity and thereby complexity (Martin et al., 2018).

The wine volatile profile is a critical point in the use of NS as starter cultures, since it is strongly dictated by the different winemaking conditions (i.e., grape must composition and winemaking practices), suggesting that several trial fermentations should be performed before adopting a novel starter NS culture in industrial production (Beckner Whitener et al., 2016; Whitener et al., 2017).

“Moschofilero” is a major Greek grapevine variety cultivated throughout Greece, but its origin and principal area of cultivation is “Mantinia” plateau in Peloponnese. Moschofilero is a noble pink-skinned aromatic variety used in the production of white fine dry wines with intense floral and fruity characters. Premium sparkling and dessert wines can be also be produced. The typical Moschofilero white wine has a lightweight, lemon color with green tinges. Intense aromas of rose and sweet grapey flavors paired with citrus and green fruits support its aromatic character. Currently, Moschofilero is of high demand in the market of PDO wines, appreciated for its refreshing, vibrant taste and fruity character. According to PDO production requirements, grape must be inoculated with selected starter yeast cultures that can express the aromatic typicity of Moschofilero. To this end, here we present for the first time means to produce terroir-driven wines with the use of Mantinia native yeasts. To enhance the varietal character and regional typicity of Moschofilero, *H. uvarum* and *L. thermotolerans*, two non-*Saccharomyces* yeast species known for high ester production, were examined in laboratory scale fermentations. *L. thermotolerans* was further selected for pilot scale vinification trials. Present results provide evidence for the combined use of indigenous yeasts in winemaking to fulfill the growing demand for wines with a sense of the place of origin, where historically developed (Vaudour et al., 2015).

## Materials and Methods

### Yeast Strains

Yeast strains *Hanseniaspora uvarum* HuMM19, *Lachancea thermotolerans* LtMM7, and *Saccharomyces cerevisiae* ScMM23 were isolated from spontaneously fermenting Moschofilero grape must from the Mantinia region, Peloponnese, Greece. Strains were previously selected based on positive enological characteristics such as ethanol and SO_2_ resistance, acetic acid production, H_2_S production and fermentation power (unpublished data). *S. cerevisiae* Zymaflore X5 (Laffort) was applied in the laboratory scale fermentations. Yeasts were identified at the species level by restriction enzyme analysis of the 5.8S-ITS rDNA region as previously described (Nisiotou et al., 2007).

### Laboratory Fermentations

Fermentations were performed in triplicate in Moschofilero grape (*Vitis vinifera* L.) must from Mantinia region [sugars 203 g/L; pH 3.31; titratable acidity 5.6 g/L, as tartaric acid; yeast assimilable nitrogen (YAN) 240 mg/L]. Fermentations were carried out at 20°C under static conditions in 1000 mL Erlenmeyer flasks containing 750 mL of pasteurized (70°C, 10 min) grape must, supplemented with 30 ppm SO_2_ in the form of potassium metabisulfite. Flasks were equipped with fermentation locks containing glycerol, permitting only CO_2_ to escape. Yeasts inocula were cultured in grape must (26°C, 18 h, 225 rpm) and added at 6 Log CFU/mL. Different inoculation schemes were applied as follows: single inoculation of the indigenous *S. cerevisiae* strain ScMM23 (IS), simultaneous inoculation (SM) of ScMM23 and *H. uvarum* HuMM19 (SMH) or *L. thermotolerans* LtMM7 (SML), sequential inoculation (SQ) of HuMM19 (SQH) or LtMM7 (SQL) followed by *S. cerevisiae* ScMM23 after *ca*. 1% vol ethanol production, and single inoculation of commercial *S. cerevisiae* (CS). Fermentation progress was monitored by following the weight loss daily.

### Pilot Scale Fermentations

*L. thermotolerans* LtMM7 and *S. cerevisiae* ScMM23 were used at pilot scale fermentations. Fermentations were carried out in triplicate in a local Mantinian winery in 250 L fermentation tanks with 150 L of Moschofilero grape must (sugars 172 g/L; pH 3.48; titratable acidity 7.12 g/L, as tartaric acid; initial YAN 71.4 mg/L) at 18°C. Potassium metabisulfite was added at 30 ppm total SO_2_. Grape must was supplemented with nitrogen by adding 40 g/hL of inactivated yeast-product before inoculation and an inactivated-yeast product containing mineral salts after 50 g/L sugar depletion. Yeast inocula were propagated in yeast extract peptone dextrose (YPD) agar at 26°C and resuspended in 1/4 strength Ringer’s solution. *L. thermotolerans* LtMM7 and *S. cerevisiae* ScMM23 were added at 6 log CFU/mL in IS, SML, and SQL inoculation schemes as described in laboratory fermentations. Spontaneous (un-inoculated) fermentations (SP) were applied in duplicate as reference. Fermentation dynamics was followed by density measurements.

### Microbiological Analysis

Must samples were taken daily, serially diluted and plated on Wallerstein laboratory nutrient agar (WL), ethanol sulfite agar (ESA), and lysine medium agar (LA) for the enumeration of total yeasts, *S. cerevisiae* and non-*Saccharomyces* species, respectively. Plates were incubated at 28°C for 2–5 days. Putative *L. thermotolerans* and *S. cerevisiae* colonies were isolated from the initial, middle and final stages of non-sterile fermentations, examined microscopically and genotyped. Genotyping of *S. cerevisiae* was performed by the interdelta region analysis with the primer set delta 12/delta 21 (Legras and Karst, 2003). For *L. thermotolerans* typing the tandem repeat-tRNA method using the primer pair TtRNASc/ISSR-MB (Barquet et al., 2012).

### Chemical Analysis

Reducing sugars, total and volatile acidity, pH, and total and free SO_2_ determinations were performed according to the methods in the Compendium of International Methods of Analysis of Musts and Wines (OIV, 2015). YAN was assayed using the formol method (Gump et al., 2001). Organic acids (citric, tartaric, malic, succinic, lactic, acetic), sugars (glucose, fructose), glycerol and ethanol were determined by HPLC according to Nisiotou et al. (2018). The major volatile compounds [acetaldehyde, ethyl acetate, methanol, 1-propanol, 2-methyl-1-propanol (Isobutanol), 3- and 2-methyl-1-butanol] of wine fermentations were determined by direct injection of wines in a gas chromatograph as previously described (Nisiotou et al., 2018). The minor volatiles of wines were determined using a headspace SPME/GC-MS method, as described by Hjelmeland et al. (2013) with slight modifications (Nisiotou et al., 2018). Peaks were quantified relative to the internal standard using peak area of an extracted ion.

### Sensory Analysis

A panel of 7 experienced assessors (3 males and 4 females, 25–55 years old, members of the Institute of Technology of Agricultural Products and of the Department of Wine, Vine and Beverage Sciences of the University of West Attica) was convened for this study. To describe the samples four aroma (tree fruits, citric fruits, floral, intensity) and seven palate (oxidation, acidity, complexity, balance, mouth aroma, persistence, after taste) terms were developed by the panel during preliminary sessions. Samples were assessed in duplicate in standard sensory analysis rooms with separate booths. Wines were presented to panelists in randomized order. The assessors scored aroma/palate attributes using a scale ranging from 0 (not perceivable) to 5 (high intensity).

### Statistical Analysis

Significant differences between chemical profiles of wines from different inoculation schemes were evaluated by Analysis of Variance (ANOVA) and Tukey’s HSD test. Principal component analysis (PCA) was applied to chemical parameters to explore relationships between samples and variables. Permutational multivariate analysis of variance (PERMANOVA) was used to compare between groups of inoculation schemes. Jaccard metric was used to calculate pairwise distances and 4,999 permutations were randomly sampled to compute F-statistics. Statistical analyses were performed with the PAST software version 3.11 (Hammer et al., 2001).

## Results

### Kinetics and Yeast Population Dynamics in Laboratory-Scale Fermentations

**FIGURE 1:**
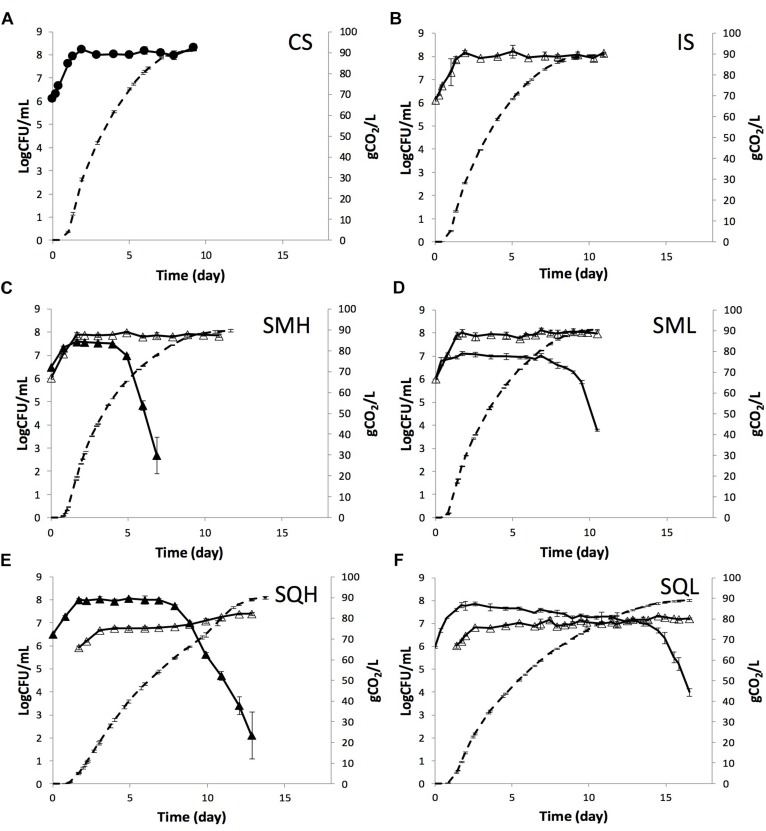
Kinetics (dashed line) and yeast population dynamics (continuous line) of laboratory fermentations performed by **(A)** commercial *S. cerevisiae* (●), **(B)** indigenous *S. cerevisiae* ScMM23 (△), **(C)** *H. uvarum* HuMM19 (▲) and *S. cerevisiae* ScMM23 (△) added simultaneously, **(D)** *L. thermotolerans* LtMM7 (−) and *S. cerevisiae* ScMM23 (△) added simultaneously, **(E)** *H. uvarum* HuMM19 (▲) and *S. c erevisiae* ScMM23 (△) added sequentially, and **(F)** *L. thermotolerans* LtMM7 (−) and *S. cerevisiae* ScMM23 (△) added sequentially.

*Hanseniaspora uvarum* HuMM19 and *Lachancea thermotolerans* LtMM7 were evaluated in pasteurized grape must. Equal quantities of each strain were added as inocula along with *S. cerevisiae* ScMM23 either simultaneously (SMH and SML inoculation schemes for HuMM19 and LtMM7, respectively, collectively called SM fermentations) or sequentially (SQH and SQL inoculation schemes, collectively called SQ fermentations). Single inoculations with the indigenous strain ScMM23 (IS) and the commercial *S. cerevisiae* Zymaflore X5 (CS) were also conducted as references. The fermentation kinetics of the different inoculation schemes are shown in Figure 1 and in Supplementary Figure S1. At the end of the fermentation courses, residual sugars were below the detection limit (<0.6 g/L) in all samples, except for SQL and SQH ferments, in which low levels of fructose were detected (Table 1). Profound differences were observed in the fermentation rate among different inoculation schemes, which was much lower in sequential than in simultaneous or single inoculations. The duration of fermentations lasted significantly longer (*P* < 0.05) in sequential additions (13.8 days for SQH and 16.5 days for SQL) than in simultaneous or single inoculations (*ca.* 11 days). The SML and IS schemes showed rather similar fermentation profiles, whereas the use of HuMM19 in SMH decreased the fermentation rate after day 4.5, thus causing an extension in the fermentation time by 1.2 days. A notable increase in the fermentation rate of SQH was observed at day 9.9, coupled with a rise in *S. cerevisiae* population by 0.6 Log CFU/mL. At this point, 72.15 g CO_2_ out of a total of 90.06 g was released.

**TABLE 1:** Chemical characteristics of wines produced in laboratory fermentations (mean ± SD, *n* = 3).

**Chemical parameter**	Inoculation protocol^*^					
	**CS**	**IS**	**SML**	**SQL**	**SMH**	**SQH**
Total acidity (as tartaric acid g/L)	8.1 ± 0.1^c^	9.2 ± 0.3^a^	8.8 ± 0.2^*ab*^	9.4 ± 0.1^a^	8.5 ± 0.4^*bc*^	7.1 ± 0.3^d^
pH	3.36 ± 0.03^a^	3.34 ± 0.01^a^	3.37 ± 0.01^a^	3.37 ± 0.01^a^	3.23 ± 0.02^b^	3.27 ± 0.03^b^
Volatile acidity (as acetic acid g/L)	0.14 ± 0.01^c^	0.13 ± 0.02^c^	0.16 ± 0.00^c^	0.28 ± 0.01^b^	0.12 ± 0.01^c^	0.36 ± 0.03^a^
Free SO_2_ (mg/L)	6.8 ± 0.8^b^	6.5 ± 1.2^b^	9.0 ± 0.0^a^	8.1 ± 0.7^*ab*^	8.1 ± 0.7^*ab*^	10.2 ± 0.0^a^
Total SO_2_ (mg/L)	12.8 ± 1.3^d^	24.7 ± 1.5^b^	26.1 ± 0.8^*ab*^	18.0 ± 0.0^c^	28.2 ± 0.0^a^	19.2 ± 1.3^c^
Citric acid (mg/L)	462 ± 17^a^	454 ± 15^*ab*^	427 ± 13^*abc*^	417 ± 13^*abc*^	407 ± 24^*bc*^	400 ± 9^c^
Tartaric acid (g/L)	2.5 ± 0.2^a^	2.5 ± 0.1^a^	2.5 ± 0.0^a^	2.4 ± 0.1^a^	2.8 ± 0.1^a^	2.6 ± 0.0^a^
Malic acid (g/L)	2.6 ± 0.1^b^	3.1 ± 0.1^a^	2.8 ± 0.1^*ab*^	2.1 ± 0.1^c^	2.5 ± 0.2^b^	2.0 ± 0.1^c^
Fructose (g/L)	<0.6	<0.6	<0.6	1.6 ± 0.2	< 0.6	1.4 ± 0.1
Succinic acid (g/L)	1.0 ± 0.1^d^	1.4 ± 0.1^*ab*^	1.3 ± 0.0^*abc*^	1.2 ± 0.0^*bcd*^	1.5 ± 0.0^a^	1.2 ± 0.0^*cd*^
Lactic acid (g/L)	<0.6	<0.6	<0.6	2.4 ± 0.1	<0.6	<0.6
Glycerol (g/L)	6.7 ± 0.3^d^	7.7 ± 0.3^*bc*^	7.6 ± 0.2^c^	7.2 ± 0.2^*cd*^	8.5 ± 0.2^*ab*^	9.1 ± 0.1^a^
Acetic acid (mg/L)	94 ± 0^*cd*^	86 ± 0^d^	113 ± 6^c^	210 ± 2^b^	97 ± 4^*cd*^	300 ± 12^a^
Ethanol (g/L)	102.7 ± 2.9^a^	100.0 ± 3.7^a^	101.2 ± 2.8^a^	96.7 ± 2.9^a^	100.7 ± 3.3^a^	98.9 ± 1.6^a^

Different letters indicate significantly different values (p < 0.05). ^*^CS, commercial S. cerevisiae; IS, S. cerevisiae ScMM23; SML, L. thermotolerans LtMM7 and S. cerevisiae ScMM23 added simultaneously; SQL, L. thermotolerans LtMM7 and S. cerevisiae ScMM23 added sequentially; SMH, H. uvarum HuMM19 and S. cerevisiae ScMM23 added simultaneously; SQH, H. uvarum HuMM19 and S. cerevisiae ScMM23 added sequentially.

The strain ScMM23 showed similar kinetics in IS and simultaneously-inoculated (SMH or SML) fermentations (Figure 1). ScMM23 peaked within 45 h after inoculation and then maintained high levels till the end of the course. A rather small (*ca.* 0.17 Log CFU/mL) albeit persistent decrease in the maximum population of ScMM23 was observed in SMH compared to IS. ScMM23 showed completely different kinetic behavior when inoculated sequentially with either HuMM19 or LtMM7. The population densities of ScMM23 in sequential fermentations were significantly lower (*ca.* 1 Log CFU/mL) compared to single or simultaneous inoculations. In SQH, a first plateau was reached within 34 h of inoculation (Figure 1E). At about day 8, the population started to gradually increase and retained these levels till the end of the fermentation. A rather small but gradual increase in the population of ScMM23 was also observed in SQL after day 11.8 till the end of the course. Notably, in both SQH and SQL fermentations, the increase in ScMM23 population coincided well with the drop of NS yeast counts. The presence of HuMM19 caused a rather small (0.2 Log CFU/mL) but continual decrease in the population of ScMM23 up to day 8, as was also observed in SMH.

As opposed to *S. cerevisiae*, the NS strains reached higher population levels in SQ than in SM fermentations. The maximum population density recorded for strain HuMM19 was by 0.47 Log CFU/mL higher in SQH than in SMH. Similarly, strain LtMM7 achieved higher density by 0.75 Log CFU/mL in SQL compared to SML. Differences were further observed in the length of the stationary phase between the different fermentation schemes. The stationary phase for both strains HuMM19 and LtMM7 lasted longer in SQ compared to SM fermentations, i.e., 7 days in SQH vs. 4 days in SMH and 15 days in SQL vs. 9 days in SML. The subsequent death rate of HuMM19 was faster in SMH than in SQH ferment, while no respective differences were observed for LtMM7 in SQL and SML ferments. HuMM19 achieved higher population densities than LtMM7 in simultaneous and sequential inoculations by 0.47 and 0.20 Log CFU/mL, respectively. Irrespective of the inoculation scheme applied, the populations of both HuMM19 and LtMM7 declined upon the release of *ca.* 60 g CO_2_ (corresponding to 8.0% vol ethanol) and 88 g CO_2_ (11.7% vol), respectively.

### Kinetics and Yeast Population Dynamics in Pilot Scale Fermentations

**FIGURE 2:**
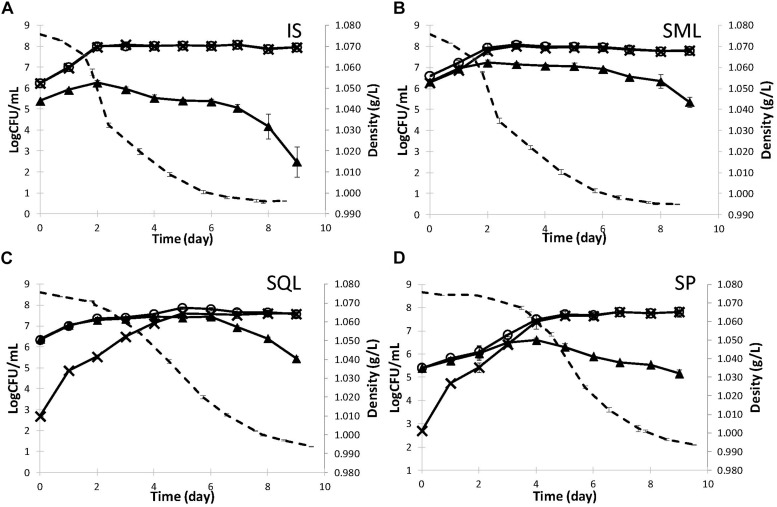
Kinetics (dashed line) and yeast population dynamics (continuous line) of pilot-scale fermentations inoculated with *S. cerevisiae* ScMM23 **(A)**, *L. thermotolerans* LtMM7/*S. cerevisiae* ScMM23 added simultaneously **(B)**, *L. thermotolerans* LtMM7/*S. cerevisiae* ScMM23 added sequentially **(C).** Spontaneous fermentation was also conducted as reference **(D)**. WL agar (○) was used for enuxmeration of total yeast population, ESA for *S. cerevisiae* (×), and LA (▲) for non-*Saccharomyces* yeasts.

Strains ScMM23 and LtMM7, which showed desirable analytical profiles in laboratory fermentations, were used in pilot scale fermentations of naturally-processed grape must at the premises of a commercial winery. The fermentation dynamics under different inoculation schemes are shown in Figure 2 and Supplementary Figure S2. IS and SM ferments showed similar fermentation kinetics, characterized by higher fermentation rates compared to SQ and spontaneous (SP) fermentations and faster completion of the course by approximately 1 day. The SP fermentation exhibited the longest lag phase followed by a sharp decline in the grape must density after day 4. Differences were observed in the growth kinetics of yeasts among the various inoculation schemes. *S. cerevisiae* ScMM23 followed similar kinetics in IS and SM fermentations, with maximum population densities of 8.10 ± 0.20 and 8.03 ± 0.07 Log CFU/mL, respectively (Figures 2A,B). The respective levels were lower in SQ (7.61 ± 0.08 Log CFU/mL) and SP (7.81 ± 0.07 Log CFU/mL) ferments (Figures 2C,D) compared to both IS and SM ferments. ScPK7 dominated in both IS and SM fermentations at percentages (93–100%). Lower percentages (14–38%) were observed in SQ fermentation while it could not be detected in SP ferment.

The addition of LtMM7 in either SM or SQ ferments significantly altered the kinetic profile of the non-*Saccharomyces* (NS) yeast fraction of the fermentative microbiota. While the indigenous NS yeasts did not exceed 6.60 ± 0.11 Log CFU/mL or 6.26 ± 0.11 Log CFU/mL in either SP or IS fermentations, significantly higher counts were observed in SQL (7.44 ± 0.18 Log CFU/mL) and SML (7.23 ± 0.15 Log CFU/mL) inoculation schemes (Figure 2). In both SM and SQ fermentations, NS populations peaked by day 2. Differences were thereafter observed, as the NS population started to gradually decline in SM, whereas in SQ it was retained at high density up to day 6. At the end of alcoholic fermentation strain LtMM7 was isolated at frequencies of 63 and 67% in SML and SQL ferment, respectively. In mixed inoculations, NS counts started to decrease at 1.021 g/L density (*ca.* 11% vol).

### The Effect of Different Inoculation Schemes on the Wine Chemical Profiles in Laboratory-Scale Fermentations

The chemical characteristics and the major volatiles (Tables 1, 2) of laboratory wines were analyzed by Permutational Multivariate Analysis of Variance (PERMANOVA). As it was shown, the inoculation scheme applied significantly affected the chemical profile of wine (*F* = 1095, *P* < 0.01). Pairwise PERMANOVA was applied to reveal the level of discrimination among the four types of inoculation schemes (Supplementary Table S1) considering *F*-values as an indicator of discrimination between samples. The chemical profile of wine was largely affected by the non-*Saccharomyces* species applied. The wine produced by the sequential inoculation using the HuMM19 strain (SQH) was the most divergent among all ferments, with *F*-values ranging from 1,368 to 2,521 followed by SMH (*F*-values 161–1,368). The time of *S. cerevisiae* addition, either simultaneously or sequentially to the NS strain, also affected the chemical profile of wines. It was shown that, irrespectively of the NS strain used, SQ-inoculated fermentations exhibited higher level of differentiation from IS as compared to SM ferments (Supplementary Table S1). The chemical profiles of IS, CS and SML schemes were more similar to each other than to other ferments.

**TABLE 2:** Major volatiles (mg/L) produced in laboratory-scale fermentations (mean ± SD, *n* = 3).

**Chemical component**	Inoculation protocol^*^					
	**CS**	**IS**	**SML**	**SQL**	**SMH**	**SQH**
Acetaldehyde	8.3 ± 0.6^c^	9.8 ± 0.9^bc^	14.2 ± 0.9^a^	12.0 ± 1.5^ab^	10.4 ± 1.5^bc^	7.7 ± 0.5^c^
Ethyl acetate	37.9 ± 0.7^d^	37.7 ± 1.0^d^	45.8 ± 0.3^d^	84.0 ± 0.7^c^	151.8 ± 4.8^b^	473.5 ± 13.0^a^
Methanol	17.3 ± 1.4^c^	18.3 ± 2.3^bc^	17.9 ± 0.6^bc^	18.4 ± 1.1^abc^	21.7 ± 0.3^a^	20.9 ± 0.6^ab^
Propanol	32.6 ± 0.8^b^	23.2 ± 0.5^d^	28.6 ± 1.2^c^	45.5 ± 1.2^a^	30.1 ± 0.3^c^	45.5 ± 0.5^a^
Isobutanol	28.5 ± 1.2^*e*^	29.2 ± 0.5^*e*^	35.7 ± 0.8^d^	70.5 ± 1.1^b^	54.3 ± 2.9^c^	84.5 ± 0.8^a^
2-Methyl-1-butanol	37.6 ± 2.9^c^	41.9 ± 1.7^abc^	39.7 ± 1.7^bc^	46.4 ± 1.8^a^	45.5 ± 0.6^a^	43.4 ± 0.8^ab^
3-Methyl-1-butanol	157.6 ± 9.2^b^	166.6 ± 4.4^ab^	166.8 ± 4.9^ab^	178.8 ± 4.9^a^	166.4 ± 1^ab^	135.0 ± 1.5^c^

Different letters indicate significantly different values (p < 0.05). ^*^CS, commercial S. cerevisiae; IS, S. cerevisiae ScMM23; SML, L. thermotolerans LtMM7 and S. cerevisiae ScMM23 added simultaneously; SQL, L. thermotolerans LtMM7 and S. cerevisiae ScMM23 added sequentially; SMH, H. uvarum HuMM19 and S. cerevisiae ScMM23 added simultaneously; SQH, H. uvarum HuMM19 and S. cerevisiae ScMM23 added sequentially.

**FIGURE 3:**
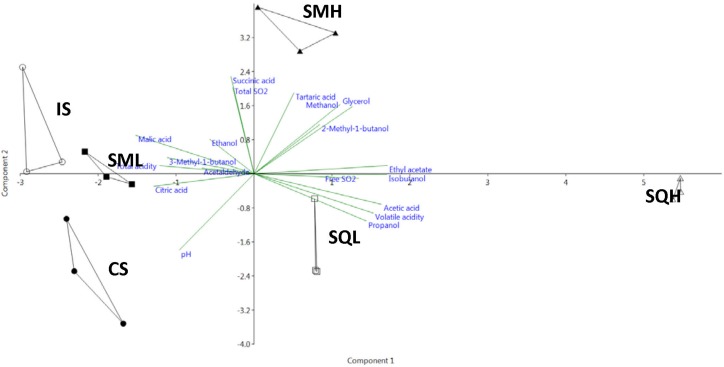
Principal Component Analysis (PCA) of the chemical attributes of the laboratory scale produced wines. PC1 and PC2 correspond to 43.4 and 22.1% of the total variation, respectively. CS, commercial *S. cerevisiae*; IS, *S. cerevisiae* ScMM23; SMH, *H. uvarum* HuMM19, and *S. cerevisiae* ScMM23 added simultaneously; SQH, *H. uvarum* HuMM19, and *S. cerevisiae* ScMM23 added sequentially; SML, *L. thermotolerans* LtMM7, and *S. cerevisiae* ScMM23 added simultaneously; SQL, *L. thermotolerans* LtMM7, and *S. cerevisiae* ScMM23 added sequentially.

The chemical profiles of the different ferments were analyzed by Principal Component Analysis (PCA) (Figure 3). The first two principal components accounted for 65.5% (43.4 and 22.1% for PC1 and PC2, respectively) of total variability. IS and SML were closely located to each other, sharing high values of malic acid which loaded negatively on PC1. On the opposite side of PC2 axis, SQH formed a distantly separated cluster showing highly positive scores on PC1 for acetic acid, ethyl acetate, isobutanol and volatile acidity. SQL was also well separated on the opposite quadrant to SQH along the PC1 axis. SMH showed high values on PCY for characteristics such as ethanol, glycerol and succinic acid.

Chemical parameters were individually subjected to ANOVA to evaluate their contribution to the differentiation of chemical profiles (Table 1). The total acidity (TA) was significantly increased in IS and SQL ferments compared to other inoculation schemes. As opposed, SQH exhibited lower TA values by more than 2 g/L. The SQ fermentations produced elevated levels of volatile acidity compared to other schemes, with SQH showing the highest amount. Residual fructose could only be detected only in SQL and SQH, whereas it was below the detection limit (<0.6 g/L) in all other ferments. A considerable amount of lactic acid was recorded in SQL (2.4 ± 0.1 g/L). The use of HuMM19 significantly enhanced the glycerol content in the respective ferments. ScMM23 yielded higher amounts of glycerol and malic acid when compared to the commercial yeast starter. The inoculation scheme was also found to affect the major volatile profile of the ferments (Table 2). The most profound difference was detected in the concentration of ethyl acetate, which was drastically increased in SQH, followed by SMH. LtMM7 caused a significant rise in ethyl acetate only in SQL; yet the level was lower than the one detected in SQH or SMH. The addition of LtMM7 caused considerable accumulations of acetaldehyde. Sequential inoculations yielded significant higher levels of ethyl acetate than simultaneous additions. Between the two NS yeasts applied, HuMM7 was strongly associated with increased production of ethyl acetate, since both SMH and SQH ferments contained significantly higher amounts than the other ferments. The addition of NS yeasts increased the levels of propanol and isobutanol. The effect was more evident in SQ than in SM ferments.

### The Effect of Different Inoculation Schemes on the Wine Chemical Profiles in Pilot-Scale Fermentations

The chemical compositions of wines produced at a commercial winery with the strains ScMM23 and LtMM7 under different inoculation schemes are shown in Tables 3–5. By applying PERMANOVA it was shown that the inoculation scheme significantly affected the chemical profiles of wines (*F* = 5.4, *P* < 0.01). As shown by pairwise PERMANOVA (Supplementary Table S1), the chemical profile of the SQL-produced wine was the most divergent among other ferments, followed by SML (mean *F*-values of 13.0 and 12.5, respectively). IS and SP ferments displayed lower mean *F*-values at 4.4 and 3.8, respectively.

**TABLE 3:** Chemical characteristics of wines produced in pilot-plant fermentations (mean ± SD, *n* = 3 or *n* = 2 in SP).

**Chemical component**	Inoculation protocol^*^			
	**IS**	**SML**	**SQL**	**SP**
Total acidity (as tartaric acid g/L)	7.0 ± 0.5^a^	6.2 ± 0.2^ab^	7.0 ± 0.0^a^	5.7 ± 0.2^b^
pH	3.27 ± 0.02^a^	3.31 ± 0.01^ab^	3.24 ± 0.01^c^	3.34 ± 0.00^b^
Volatile acidity (as acetic acid g/L)	0.14 ± 0.02^a^	0.14 ± 0.01^a^	0.16 ± 0.01^a^	0.15 ± 0.00^a^
Citric acid (g/L)	464 ± 33^a^	421 ± 51^a^	472 ± 20^a^	467 ± 38^a^
Tartaric acid (g/L)	3.2 ± 0.3^a^	2.9 ± 0.1^a^	3.0 ± 0.2^a^	3.2 ± 0.0^a^
Malic acid (g/L)	2.9 ± 0.3^a^	2.5 ± 0.3^a^	2.4 ± 0.1^a^	2.5 ± 0.1^a^
Lactic acid (g/L)	0.2 ± 0.0^b^	0.2 ± 0.0^b^	1.9 ± 0.1^a^	0.1 ± 0.0^b^
Succinic acid (g/L)	0.8 ± 0.2^a^	0.6 ± 0.1^a^	0.7 ± 0.0^a^	0.5 ± 0.0^a^
Glycerol (g/L)	5.5 ± 0.8^a^	4.7 ± 0.5^a^	6.0 ± 0.3^a^	5.3 ± 0.4^a^
Acetic acid (g/L)	62 ± 15^a^	57 ± 14^a^	69 ± 9^a^	52 ± 16^a^
Ethanol (g/L)	72.7 ± 3.9^a^	67.5 ± 10.9^a^	76.0 ± 2.6^a^	77.0 ± 6.6^a^

Different letters indicate significantly different values (p < 0.05). ^*^IS, S. cerevisiae ScMM23; SML, L. thermotolerans LtMM7 and S. cerevisiae ScMM23 added simultaneously; SQL, L. thermotolerans LtMM7 and S. cerevisiae ScMM23 added sequentially; SP, Spontaneous fermentation.

**TABLE 4:** Major volatiles (mg/L) of wines produced in pilot scale fermentations (mean ± SD, *n* = 3 or *n* = 2 in SP).

**Chemical component**	Inoculation protocol^*^			
	**IS**	**SML**	**SQL**	**SP**
Acetaldehyde	40.8 ± 15.0^a^	35.1 ± 7.6^a^	107.0 ± 21.8^b^	48.8 ± 18.1^a^
Ethyl acetate	27.8 ± 6.3^a^	36.3 ± 4.9^a^	58.0 ± 6.7^a^	61.9 ± 27.6^b^
Methanol	40.1 ± 1.5^a^	37.6 ± 4.6^a^	43.2 ± 2.0^a^	41.1 ± 3.9^a^
Propanol	20.9 ± 2.5^a^	19.9 ± 2.0^a^	32.8 ± 1.2^b^	25.9 ± 4.4^ab^
Isobutanol	17.5 ± 2.4^a^	15.5 ± 2.1^a^	24.3 ± 1.0^b^	18.5 ± 2.9^ab^
2-Methyl-1-butanol	22.5 ± 3.4^a^	16.5 ± 2.2^a^	19.5 ± 0.3^a^	20.5 ± 2.6^a^
3-Methyl-1-butanol	113.5 ± 10.2^a^	101.1 ± 13.1^a^	121.6 ± 2.5^a^	114.8 ± 9.4^a^

Different letters indicate significantly different values (p < 0.05).^*^IS, S. cerevisiae ScMM23; SML, L. thermotolerans LtMM7 and S. cerevisiae ScMM23 added simultaneously; SQL, L. thermotolerans LtMM7 and S. cerevisiae ScMM23 added sequentially; SP, Spontaneous fermentation.

**TABLE 5:** Minor volatiles (μg/L)^*^ of wines produced in pilot scale fermentations (mean ± SD, *n* = 3 or *n* = 2 in SP).

**Chemical component**	**Inoculation protocol^∗∗^**			
	**IS**	**SML**	**SQL**	**SP**
**ESTERS**				
Methyl acetate	54 ± 11^a^	49 ± 9^a^	61 ± 10^*ab*^	89 ± 18^b^
Propyl acetate	76 ± 28^a^	92 ± 15^a^	171 ± 31^b^	116 ± 36^*ab*^
Isobutyl acetate	193 ± 97^a^	239 ± 55^*ac*^	442 ± 110^*bc*^	251 ± 47^*ac*^
Isoamyl acetate	14,590 ± 2,645^a^	14,238 ± 1,406^a^	20,049 ± 4,186^a^	14,232 ± 1,908^a^
Hexyl acetate	691 ± 154^a^	738 ± 139^a^	1,239 ± 210^b^	1,889 ± 153^c^
2-Phenylethyl acetate	1,211 ± 155^a^	948 ± 77^a^	2,140 ± 124^b^	2,640 ± 597^b^
Ethyl propanoate	130 ± 14^*ab*^	83 ± 17^b^	169 ± 40^a^	87 ± 42^*ab*^
Ethyl butanoate	451 ± 102^a^	535 ± 105^a^	621 ± 115^a^	576 ± 80^a^
Ethyl hexanoate	16,316 ± 2,151^a^	16,332 ± 1,620^a^	14,210 ± 1,631^a^	18,087 ± 1,005^a^
Ethyl heptanoate	58 ± 13^a^	55 ± 4^a^	52 ± 9^a^	66 ± 12^a^
Ethyl lactate	86 ± 33^a^	64 ± 9^a^	694 ± 73^b^	29 ± 0^a^
Methyl octanoate	375 ± 42^a^	299 ± 12^*ac*^	228 ± 28^*bc*^	371 ± 110^a^
Ethyl octanoate	166,375 ± 65,723^a^	212,081 ± 7,727^a^	130,709 ± 12,136^a^	223,679 ± 56,930^a^
Isoamyl hexanoate	360 ± 48^a^	633 ± 76^b^	343 ± 45^a^	505 ± 89^*ab*^
Ethyl nonanoate	86 ± 21^a^	116 ± 7^a^	92 ± 10^a^	105 ± 26^a^
Methyl decanoate	88 ± 5^a^	180 ± 22^b^	124 ± 15^a^	134 ± 25^*ab*^
Ethyl decanoate	68,583 ± 11,992^c^	180,151 ± 25,322^a^	112,771 ± 15,524^*bc*^	149,481 ± 25,019^*ab*^
3-Methylbutyl octanoate	2,584 ± 735^a^	4,686 ± 394^b^	2,415 ± 105^a^	4,851 ± 884^b^
Diethyl butanedioate	92 ± 27^a^	81 ± 19^*ab*^	37 ± 6^b^	32 ± 3^b^
Ethyl 9-decenoate	6,967 ± 3,356^a^	9,489 ± 4,412^*ab*^	6,320 ± 702^a^	19,773 ± 6,803^b^
Ethyl dodecanoate	11,805 ± 5,577^a^	22,544 ± 4,339^a^	12,531 ± 2,474^a^	24,091 ± 7,733^a^
Ethyl tetradecanoate	224 ± 111^a^	263 ± 99^a^	366 ± 16^a^	275 ± 125^a^
Ethyl hexadecanoate	216 ± 19^a^	87 ± 22^a^	187 ± 1^b^	177 ± 56^b^
**ALCOHOLS**				
1-Butanol	22 ± 18^a^	12 ± 1^a^	27 ± 1^a^	18 ± 3^a^
1-Hexanol	344 ± 18^a^	297 ± 20^a^	423 ± 20^b^	300 ± 51^a^
2,3-Butanediol (isomer 1)	185 ± 52^a^	119 ± 31^a^	152 ± 51^a^	197 ± 59^a^
2,3-Butanediol (isomer 2)	47 ± 5^a^	27 ± 9^a^	43 ± 9^a^	77 ± 19^b^
1-Decanol	35 ± 14^a^	54 ± 11^*ab*^	68 ± 6^b^	82 ± 3^b^
Phenylethyl Alcohol	2,719 ± 643^a^	2,248 ± 163^a^	2,863 ± 482^a^	2,495 ± 256^a^
**ACIDS**				
Acetic acid	169 ± 27^a^	152 ± 24^a^	221 ± 42^a^	132 ± 31^a^
Hexanoic acid	302 ± 24^a^	419 ± 19^*ac*^	512 ± 66^*bc*^	445 ± 127^*ac*^
Octanoic acid	1,096 ± 337^a^	1,357 ± 130^a^	1,803 ± 311^a^	1,692 ± 261^a^
Decanoic acid	309 ± 216^a^	891 ± 322^a^	921 ± 233^a^	539 ± 14^a^
**OTHER COMPOUNDS**				
b-Citronellol	87 ± 13^a^	70 ± 13^a^	83 ± 6^a^	66 ± 4^a^
Geraniol	88 ± 29^a^	97 ± 12^a^	111 ± 8^a^	92 ± 9^a^
b-Damascenone	30 ± 9^a^	29 ± 2^a^	52 ± 3^b^	34 ± 5^a^
1,1-Diethoxy ethane	539 ± 90^a^	367 ± 70^a^	1,805 ± 500^b^	610 ± 328^a^

Different letters indicate significantly different values (p < 0.05). ^*^Concentrations relative to internal standard (3-pentanol). Expressed as the ratio of each compound peak area to that of internal standard multiplied by its concentration (1000 μg/L). ^∗∗^IS, S. cerevisiae ScMM23; SML, L. thermotolerans LtMM7, and S. cerevisiae ScMM23 added simultaneously; SQL, L. thermotolerans LtMM7, and S. cerevisiae ScMM23 added sequentially; SP, Spontaneous fermentation.

**FIGURE 4:**
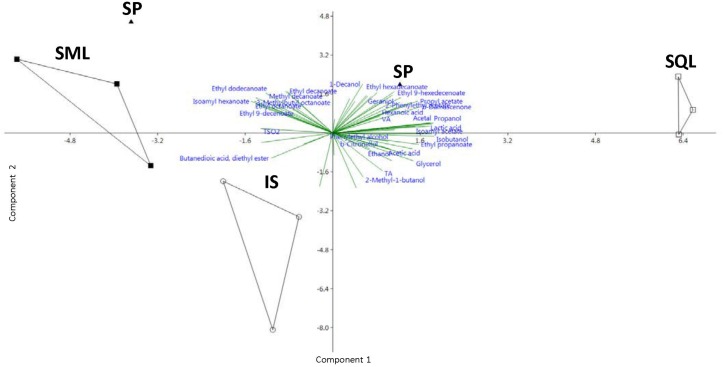
Principal Component Analysis (PCA) of the chemical attributes of the pilot scale produced wines. PC1 and PC2 correspond to 35.7 and 22.4% of the total variation, respectively. IS, *S. cerevisiae* ScMM23; SML, *L. thermotolerans* LtMM7, and *S. cerevisiae* ScMM23 added simultaneously; SQL, *L. thermotolerans* LtMM7, and *S. cerevisiae* ScMM23 added sequentially; SP, Spontaneous fermentation.

The chemical profiles of different ferments were compared by PCA (Figure 4). The cumulative variability for the first two components was at 58.1% (35.7 and 22.4% for PC2). The profiles of SQL ferments were the most distantly located on the PCA plot, exhibiting highly positive scores on PC1 for numerous characteristics, such as acetic acid, damascenone, isoamyl acetate and phenylethyl alcohol. IS and SML ferments were separated from SQL along the PC2 direction. IS was valued negatively on PC2, mainly due to the presence of malic acid, succinic acid and 2-methyl-1-butanol. The SML ferment exhibited high values of ethyl esters that loaded negatively on the PC1, such as ethyl octanoate, isoamyl hexanoate, methyl decanoate, ethyl decanoate and 3-methylbutyl octanoate. SP ferments were most closely located to SML, due to the high values of certain compounds on PC2, such as ethyl esters (Hexyl acetate, Ethyl hexanoate, Ethyl 9-decenoate) or alcohols (1-Decanol and 2,3-Butanediol).

Each of the chemical parameters was subjected to ANOVA to investigate its contribution to the chemical profile of wines. Total acidity was significantly lower (*P* < 0.05) in SP compared to IS and SQL ferments (Table 3). The use of LtMM7 in SQL fermentation caused the decline of pH (*P* < 0.05) probably due to the production of considerable amounts of lactic acid. Significant raises were also observed in acetaldehyde, propanol and isobutanol levels in SQL ferments. Differences were further observed in the concentration of minor volatiles, mostly associated with the use of LtMM7 in the SQL inoculation scheme (Table 5). LtMM7 notably increased the levels of acetate esters, especially in SQL ferment in which their concentration was twofold higher compared to IS. Propyl acetate, isobutyl acetate, hexyl acetate and 2-phenylethyl acetate were significantly higher in SQL than in IS or SML. LtMM7 was also associated with significantly higher levels of ethyl esters when used in SML inoculation. The most striking differences were observed in the levels of ethyl decanoate, 3- methylbutyl octanoate and isoamyl hexanoate. The concentration of total acids and terpenes were also significantly affected by the addition of LtMM7, with SQL ferment exhibiting the highest values.

### Sensory Analysis

**FIGURE 5:**
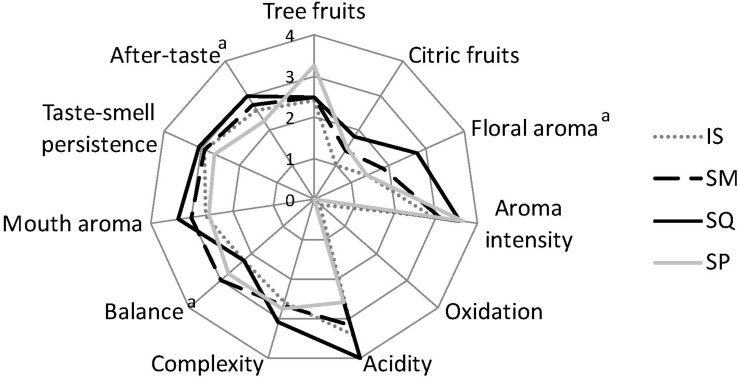
Means of the sensory attributes for the pilot scale produced wines. IS, *S. cerevisiae* ScMM23; SML, *L. thermotolerans* LtMM7, and *S. cerevisiae* ScMM23 added simultaneously; SQL, *L. thermotolerans* LtMM7, and *S. cerevisiae* ScMM23 added sequentially; SP, spontaneous fermentation. Significant differences among samples are indicated by superscript (*p* < 0.05).

Figure 5 shows the mean scores of sensory characteristics of Moschofilero wines as evaluated by the sensory assessors’ panel. According to ANOVA, significant differences (*p* < 0.05) among samples were detected for three descriptors, i.e., floral aroma, balance, and after-taste. In particular, SQL wine was characterized by the highest intensity of floral aroma and after-taste, followed by SML, while SML was the most balanced among all the wines. Considering multiple pairwise comparisons, SP wine showed significantly higher intensity of tree fruit aroma than the other wines. SP was also characterized by the highest overall aroma intensity (significantly different from IS). SQL wines were found to have the most intense citric fruit aroma and complexity, which were significantly different from IS wine. In addition, SQL wines had increased acidity and palate complexity (significantly higher compared to IS), but a lower balance as compared to other wines.

## Discussion

“Moschofilero” is a native Greek grape variety traditionally cultivated in the “Mantinia” plateau in Peloponnese, Greece, since ancient times. It is used in the production of “Mantinia” PDO *blanc de gris* white wines with a characteristic fruity and floral aromatic profile (rose, lemon, and jasmine) that has been associated with its place of origin. To enhance the connection of Moschofilero wines with its original area of cultivation, the introduction of the notion of “microbial terroir” was considered in the present study, by applying autochthonous yeasts as starters in the fermentation of grape must. Besides, high ester producing species were considered for their potential to enhance the floral aromas and fruity bouquet of wines.

Here, two autochthonous *H. uvarum* and *L. thermotolerans* strains were investigated for their ability to contribute the aromatic features associated with the wild yeast flora, along with a *S. cerevisiae* strain, also isolated from the Mantinia vineyard, to complete the fermentation course. The fermentation kinetics in sterile must was affected by both the inoculation scheme and the NS yeast species applied. When simultaneously inoculated, *S. cerevisiae* was highly antagonistic and reduced the population of both NS yeasts as compared to SQ inoculations. This is commonly attributed to higher sugar fermentation capacity and nitrogen uptake of *S. cerevisiae* compared to several NS wine yeasts including *H. uvarum* (Andorrà et al., 2012; Albergaria and Arneborg, 2016). The population reduction was larger for *L. thermotolerans* than *H. uvarum*, showing that the former species might be more susceptible to *S. cerevisiae* activity. *L. thermotolerans* has been shown to immediately diminish upon addition of *S. cerevisiae* possibly through a concerted action of cell-to-cell contact and antimicrobial peptides (Benito et al., 2015, 2016; Kemsawasd et al., 2015). In some other studies, though, *L. thermotolerans* showed increased persistence in mixed fermentations, especially in SQ inoculations (Comitini et al., 2011; Gobbi et al., 2013). These results together suggest that the viability of NS yeasts in mixed-culture or spontaneous fermentations may not be solely defined at the species level but is also highly strain-dependent (Wang et al., 2015). It is important thus that strain compatibilities should be considered upon designing of mixed inocula in wine fermentations.

The wine chemical profile in sterile must fermentations was highly differentiated by applying the sequential inoculation scheme. The strongest differentiation was associated with the use of *H. uvarum*, despite the fact that it showed much lower persistence than *L. thermotolerans*. However, its presence was strongly correlated with significant increase of acetic acid and ethyl acetate. *H. uvarum* often generates elevated levels of acetate and ethyl acetate (du Toit and Pretorius, 2000; Ciani et al., 2006). Nevertheless, it is an important biotic component of wine fermentation as a high producer of fruity esters, while its low frequency has been associated with reduced aroma complexity of wine (Rementeria et al., 2003). Nevertheless, there is great genetic and phenotypic variability among vineyard-associated *H. uvarum* isolates. For instance, the use of a selected *H. uvarum* isolate increased medium chain fatty acid (MCFA) ethyl ester content without raising the acetate concentration beyond the acceptable limit (Hu et al., 2018). In the present study, although *H. uvarum* strain HuMM19 produced relatively low levels of acetic acid (according to the optimal concentration range of 0.2–0.7 g/L) (Lambrechts and Pretorius, 2000), it strongly increased the concentration of ethyl acetate far above the acceptable limits (100 mg/L), responsible for solvent/nail polish-like odor (Sumby et al., 2010). On the other hand, the use of *L. thermotolerans* was associated with several positive enological characteristics but not any obvious defects, and was thus selected to perform further fermentations at pilot scale trials.

Ester production is an important quality attribute of yeast activity, contributing significantly to the aroma of wines (Ugliano and Henschke, 2009). The total amount as well as the profile of ester production shows high variability depending on the yeast species or strains implicated, shaping thereby wine style and character (Beckner Whitener et al., 2016). Although *L. thermotolerans* was one of the first NS yeasts to be released commercially as starter culture for winemaking, the profile of ester production was only recently explored in more detail (Beckner Whitener et al., 2016; Whitener et al., 2017), while very few studies have investigated its performance in industrial scale fermentations (Gobbi et al., 2013). It has been generally accepted that *L. thermotolerans* affects the aroma profile of wines by producing several acetate esters rather than ethyl esters (Morales et al., 2017; Morata et al., 2018). When compared to *S. cerevisiae*, it generally produces significantly lower levels of acetate esters or total esters, excluding ethyl lactate and ethyl acetate (Gobbi et al., 2013; Balikci et al., 2016; Beckner Whitener et al., 2016; Whitener et al., 2017). Here the strain LtMM7 significantly increased the levels of both acetate esters and ethyl esters. It is also important to note that the inoculation protocol applied significantly affected the relative production of acetate esters and ethyl esters. In particular, acetate esters such as isobutyl acetate, hexyl acetate and 2-phenylethyl acetate, which impart fruity or floral aromas, were significantly increased in the SQL ferment. Importantly, the level of 2-phenylethyl acetate, which confers floral, rosy and honey-like with fruity nuance odors, all typical varietal aromas of Moschofilero, was doubled in SQL compared to IS. In line with that, the floral aroma intensity was significantly higher in SQL wine compared to other wines. On the other hand, higher levels of total ethyl esters were associated with SML (eight different odorant active compounds). The most striking differences were observed in the levels of ethyl decanoate (soapy, floral), 3-methylbutyl octanoate (fruit) and isoamyl hexanoate (anise, fruit, spice). The increase in the concentration of ethyl esters positively correlates with the fruity aroma of wine (Hu et al., 2018) and the respective wines were characterized by higher overall aroma intensity and mouth aroma than IS. Several fermentation factors have been evaluated to stimulate ethyl ester production, such as the addition of MCFA precursors (Saerens et al., 2008), nitrogen additions (Rollero et al., 2015) or mixed fermentations with NS yeasts. With respect to the later, accumulated data show that ethyl ester content can be enhanced by the use of NS yeasts in a strain-specific way (Hu et al., 2018; Nisiotou et al., 2018).

Varietal aromatic precursors, such as terpenes and C13-norisoprenoids, are predominantly found in grapes in their glycosylated odorless form. They can then be hydrolyzed by glucosidases to free aromatic derivatives during fermentation. Moschofilero is an aromatic (floral) variety rich in total terpenes, a large portion of which is in bound form (Metafa and Economou, 2013). Geraniol constitutes the highest fraction of bound terpenes also accounting for about half of the total free terpenic content in wine (Metafa and Economou, 2013). It seems that a fermentation protocol which can liberate bound aromatic compounds could enhance the varietal character of Moschofilero. Contrary to previous beliefs (Comitini et al., 2011), current studies show that certain strains of *L. thermotolerans* may exhibit high β-glucosidase activity (Cordero-Bueso et al., 2012). Recently, *L. thermotolerans* was found to increase the free terpenic content (farnesol, geraniol, α-ionene, and cosmene) in Sauvignon blanc wines (Beckner Whitener et al., 2016). In another study, *L. thermotolerans* was shown to produce the highest relative concentration of linalool than other yeast species (Whitener et al., 2017). In the present study, the use of *L. thermotolerans* in SQL fermentation was shown to increase the concentrations of geraniol and damascenone. Geraniol, a major odor compound of Moschofilero, has a low odor threshold (30 μg/l) (Guth, 1997) and is associated with several typical aromas of Moschofilero, such as floral, sweet, rosy, fruity and a citrus nuance. β-damascenone is a key odor in grapes with low odor threshold (4–7 μg/L in wine matrix) (Pineau et al., 2007). The contribution of damascenone in wine flavor is important either directly by conferring floral and exotic fruit notes (Guth, 1997) or indirectly by strengthening the fruit aromas of other compounds (Pineau et al., 2007).

## Conclusion

In conclusion, the present results show that the use of an indigenous *L. thermotolerans* strain as a NS yeast starter along with a selected *S. cerevisiae* strain may enhance the typical floral and fruity aromas of Moschofilero, one of the most important Greek white wines. This is the first study to show a significant increase in ethyl ester fraction by *L. thermotolerans*. Importantly, the inoculation scheme significantly affected the relative production of acetate esters and ethyl esters. As opposed, a selected indigenous *H. uvarum* strain did not show a prominent enological potential to be used as a fermentation starter, coinciding with previous studies showing that this NS species may generate elevated levels of ethyl acetate. Taken together, well-selected indigenous NS strains may be used in multispecies fermentations to enhance the organoleptic properties and the typicity of local wines by introducing the so-called microbial *terroir* effect.

## Data Availability

All datasets generated for this study are included in the manuscript and/or the Supplementary Files.

## Author Contributions

AN designed the study, performed the experiments, analyzed the data, and wrote the manuscript. AM conducted the experiments and analyzed the data. CT contributed to data analysis and writing the manuscript. GB analyzed the data and contributed to writing the manuscript.

## Conflict of Interest Statement

The authors declare that the research was conducted in the absence of any commercial or financial relationships that could be construed as a potential conflict of interest.
