# Supplementary material for: Indigenous Yeast Interactions in Dual-Starter Fermentations May Improve the Varietal Expression of Moschofilero Wine
Source: Front Microbiol. 2019 Jul 26;10:1712. doi: 10.3389/fmicb.2019.01712 (PMC6677089; doi:10.3389/fmicb.2019.01712)
Supplement: Supplementary file 1 [file Data_Sheet_1.docx]

Supplementary Material

# Supplementary Figures and Tables

## Supplementary Figures

**Figure S1.** Kinetics of laboratory-scale fermentations inoculated with indigenous *S. cerevisiae* ScMM23 (IS), commercial *S. cerevisiae* (CS), *H. uvarum* HuMM19 and *S. cerevisiae* ScMM23added simultaneously (SMH) or sequentially (SQH), and *L. thermotolerans* LtMM7 and *S. cerevisiae* ScMM23 added simultaneously (SML) or sequentially (SQL).

**Figure S2.** Kinetics of pilot-scale fermentations inoculated with indigenous *S. cerevisiae* ScMM23 (IS), *L. thermotolerans* LtMM7 and *S. cerevisiae* ScMM23 added simultaneously (SML) or sequentially (SQL). Spontaneous fermentation (SP) was also conducted as reference.

**Supplementary Table S1.** F-values of pair wise PERMANOVA corresponding to different inoculation schemes at laboratory- and pilot-levels.

| Inoculation scheme 1 | Inoculation scheme 2 | F-statistic |
| --- | --- | --- |
| Laboratory-scale fermentations | | |
| CS | IS | 6.2 |
| CS | SMH | 254.3 |
| CS | SQH | 1991.0 |
| CS | SML | 8.5 |
| CS | SQL | 87.4 |
| IS | SMH | 384.1 |
| IS | SQH | 2501.0 |
| IS | SML | 5.3 |
| IS | SQL | 135.3 |
| SMH | SQH | 1368.0 |
| SMH | SML | 363.6 |
| SMH | SQL | 161.4 |
| SQH | SML | 2521.0 |
| SQH | SQL | 2041.0 |
| SML | SQL | 107.3 |
| Pilot-scale fermentations | | |
| IS | SML | 8.4 |
| IS | SQL | 2.0 |
| IS | SP | 2.8 |
| SML | SQL | 29.3 |
| SML | SP | 0.8 |
| SQL | SP | 7.8 |
